# Neural Correlates of Perceiving Emotional Faces and Bodies in Developmental Prosopagnosia: An Event-Related fMRI-Study

**DOI:** 10.1371/journal.pone.0003195

**Published:** 2008-09-17

**Authors:** Jan Van den Stock, Wim A. C. van de Riet, Ruthger Righart, Beatrice de Gelder

**Affiliations:** 1 Laboratory of Cognitive and Affective Neuroscience, Tilburg University, Tilburg, The Netherlands; 2 Old Age Psychiatry Department, University Hospitals Leuven, Leuven, Belgium; 3 Martinos Center for Biomedical Imaging, Massachusetts General Hospital, Charlestown, Massachusetts, United States of America; University of Minnesota, United States of America

## Abstract

Many people experience transient difficulties in recognizing faces but only a small number of them cannot recognize their family members when meeting them unexpectedly. Such face blindness is associated with serious problems in everyday life. A better understanding of the neuro-functional basis of impaired face recognition may be achieved by a careful comparison with an equally unique object category and by a adding a more realistic setting involving neutral faces as well facial expressions. We used event-related functional magnetic resonance imaging (fMRI) to investigate the neuro-functional basis of perceiving faces and bodies in three developmental prosopagnosics (DP) and matched healthy controls. Our approach involved materials consisting of neutral faces and bodies as well as faces and bodies expressing fear or happiness. The first main result is that the presence of emotional information has a different effect in the patient vs. the control group in the fusiform face area (FFA). Neutral faces trigger lower activation in the DP group, compared to the control group, while activation for facial expressions is the same in both groups. The second main result is that compared to controls, DPs have increased activation for bodies in the inferior occipital gyrus (IOG) and for neutral faces in the extrastriate body area (EBA), indicating that body and face sensitive processes are less categorically segregated in DP. Taken together our study shows the importance of using naturalistic emotional stimuli for a better understanding of developmental face deficits.

## Introduction

Recognizing faces of family and friends usually proceeds effortlessly. Yet a minority of people has difficulties telling apart who they are meeting with or remembering who they met previously when they can only go by the visual memory of the face. These problems can be quite dramatic, even to the point where they fail to recognize the face of their own spouse or child or for that matter their own face. The original reports of face recognition deficits for which the term prosopagnosia [1] was coined concerned cases of brain damage sustained in adulthood. More recently there have been reports of face recognition deficits that do not appear to be associated with any known neurological history. Although there are still only a few systematic reports of this condition, many more cases are described now compared to a decade ago and some authors have argued that as much as 2% of the population suffers from face recognition difficulties [2]. In analogy with developmental dyslexia these cases are now commonly referred to as developmental prosopagnosia (DP), referring to the possible origin of the adult face recognition deficit in anomalous development of the full face recognition skills. This behavioral deficit may include an anomaly in the putative congenital basis involved in the acquisition of the skill, but so far very little is known about this genetic basis and its importance for explaining behavioral deficits [3].

Recent research on behavioral face recognition deficits and their neural basis has followed the leads from the reports on the neural basis of face recognition in normals as mainly revealed in fMRI studies over the last decade. There is now a consensus in the literature that face recognition is implemented in a network of brain areas [4], [5]. Among these, an area in the fusiform gyrus (FG), labeled the fusiform face area (FFA) [6], [7], has attracted most attention. Next to this area, the role of the inferior occipital gyrus (IOG) is repeatedly stressed in normal e.g. [8]–[10] and anomalous face recognition [11]. But it is fair to say that the functional significance of these two main areas for person recognition and its deficits is not yet entirely clear.

Investigations of the neuro-functional correlates of DP with fMRI have yielded inconsistent results [11]–[16] (see Table 1 for an overview). The first fMRI-study including a DP case by Hadjikhani and de Gelder [11] found no face-specific activation in these two areas. A similar pattern was observed with another DP case [15]. On the other hand, other studies reported normal face-specific activation in developmental prosopagnosics (DPs) despite their severe behavioral deficits in face recognition [12]–[14], [16]. These findings suggest that intact functioning of the FFA and IOG are necessary, but not sufficient for successful face recognition.

**Table 1:** Results from fMRI-studies on prosopagnosia.

Studies with developmental prosopagnosics						
	N	Lesion localisation	task	comparison	result	
					FFA	IOG
Hadjikhani & de Gelder (2002)	1	n.a.	Passive viewing	Faces>objects	−	−
				Faces>houses	+	−
Hasson et al. (2003)	1	n.a.	One back	Faces>buildings	+	+
Avidan et al. (2005)	4	n.a.	One back	Faces>(buildings & objects)	+	+
Bentin et al. (2007)	1	n.a.	Oddball	Faces>places	−	−
				Faces>objects	−	−
Degutis et al. (2007)^1^	1	n.a.	One back	Faces>scenes	+	+
Williams et al. 2007	1	n.a.	One back	Faces>scenes	+	
Van den Stock et al.	3	n.a.	Oddball	(Emotional+neutral faces)>houses	decreased neutral face activity	+

Studies with acquired prosopagnosics							
	N	Lesion localisation	task	comparison	result		
					FFA		IOG
Marotta et al. (2001)	2	SM: right temporal	oddball	Faces>objects	SM	(+)	
		CR: right temporal			CR	−	
Hadjikhani & de Gelder (2002)	2	No evident lesions	Passive viewing	Faces>objects	−		−
				GA: Faces>scramble	−		−
				RP: Faces>houses	−		+
Rossion et al. (2003)	1	Right posterior inferior occipital to posterior fusiform gyrus; left middle fusiform gyrus	One back	Faces>objects	+		
Steeves et al. (2006)	1	Bilateral lateral occipital; left superior parieto-occipital sulcus	Oddball	Faces>scenes	+		
Sorger et al. (2007)	1	Right ventral occipito-temporal; right middle temporal gyrus; left FG, IOG and lingual gyrus; left medial cerebellum (see also Rossion et al. 2003)	One back	Faces>objects	+		(l)

1 This study reports about a training program administered to a patient. We report the fMRI result preceding the training.

Abbreviations: n.a.: not applicable; FFA: Fusiform Face Area; FG: Fusiform Gyrus; IOG: Inferior Occipital Gyrus; SM, CR, GA & RP refer to subjects; +: significant activation; −: no significant activation; (l−): only left activation is observed.

In view of the many different kinds of information a face provides (gender, age, emotion, familiarity, attractiveness etc.) and the different ways in which this information is called upon and used in daily life (whether the context only requires rapid detection that there is a face present, or on the contrary, full recognition of all facial attributes including name retrieval), it is worth stressing that the contextual requirements and the task settings are very important for evaluating face recognition problems and for understanding its neuro-functional basis and possible deficits. A finely tuned comparison of face recognition skills with other object recognition skills at the behavioral and neuro-functional level requires comparable task settings whether the object categories to be matched are faces or any other category that is suitable [17]–[21]. Since faces convey many different kinds of information it has so far been a daunting task to find a matching category to use as control stimuli. Previous approaches to find the best matching category have tended to explore either the physical similarity dimension (for example, using a continuum of more or less face like stimuli), the perceptual one or the functional one (for example, expertise with one or another specific object category). This has fed an ongoing debate about whether face processing mechanisms are qualitatively different from the processing mechanisms for objects (modularity hypothesis) [22], or on the other hand whether relative face specificity reflects the level of perceptual expertise with the stimulus category (expertise hypothesis) [9], [23]. As a matter of fact there are very few objects other than faces for which strong claims about category specific representation have been made. One exception concerns houses. Several studies report that this object category differentially activates a region around the collateral sulcus [24]–[26].

An interesting object category not used so far concerns human bodies. Recently, it has been shown in normal subjects that perceiving human bodies or body parts activates an area in extrastriate cortex, labeled extrastriate body area (EBA) [27]. More recently a second body specific area was defined in the FG [28], [29]. This body sensitive area in FG overlaps at least partially with the face-sensitive one and it has been termed the fusiform body area (FBA). In parallel, recent findings show that the close similarities between face and body perception exist at the level of perceptual mechanisms as revealed by the inversion effect (a decline in performance for inverted stimuli compared to upright stimuli that is more pronounced for faces than for other object categories [30]), since the same inversion effect has been reported for bodies [31], [32] for reviews, see [33], [34].

These behavioral and neuro-functional similarities between perceiving faces and bodies in normals and the fact that bodies represent a distinct but yet very closely related object category, raise the issue how bodies are processed in DP. A study by Duchaine et al. [35] presented natural faces and computer generated neutral body postures for testing face and body identity recognition in a DP patient using a sequential identity matching paradigm involving a minimal memory component. The performance of the patient was impaired for the faces, but within normal range for the bodies suggesting dissociation between face and body processing mechanisms with these task settings. Another study used event-related potentials (ERP) to investigate face and body perception in four DPs and found abnormal brain activation in the early time windows of the EEG (around 170 ms) for both faces and bodies in three of the four DPs [36].

A second main objective of the present study is to investigate how the neural underpinnings of face and body processing in prosopagnosia are influenced by emotional information in the face and the body. As a matter of fact, the face-sensitive area in FG is well known from investigations of face recognition using neutral faces but it also figures predominantly in research on the neural basis of recognizing facial expressions. The presence of an emotion expression adds realism to the face but may also be an interesting developmental factor. Studies with younger subjects have predominantly reported higher activation for fearful faces, compared to neutral faces [37]–[41], but a recent study with both adolescents and adults found a reverse pattern in the FFA, namely higher activation for neutral than for fearful faces [42]. The mechanism of this emotional modulation in the FFA may be based on feedback loops with the amygdala [39], [41]. A similar explanation has been proposed for the increased activation in FG sensitive to body images representing an emotional expression [28].

So far, the evidence concerning the neural correlates of processing emotional faces in DP is scarce. One study by de Gelder et al. [43] investigated this issue in acquired prosopagnosics (prosopagnosia occurring after brain damage). The included patients had lesions in either the FG, IOG or both. The results showed that the patients more strongly activated other face sensitive areas like the superior temporal sulcus (STS) or amygdala when they perceive facial expressions compared to neutral faces. The patients were also more accurate and faster in processing emotional faces compared to neutral faces, a finding that has been reported previously [44]–[46]. Since the patients in de Gelder et al. [43] had lesions in the ventral occipito-temporal cortex, the question arises how these brain areas respond to emotional information in prosopagnosics with severe face recognition problems but no known brain anomalies. To investigate this issue we presented the participants with neutral, fearful and happy facial and bodily expressions.

## Methods

### Participants

The DPs were recruited after they had contacted us via our website or through reports in the popular press. All participants report life-long problems in recognizing people and typically complain about difficulties when meeting familiar persons unexpectedly and the ensuing social problems. AM (female) is a 54-year old housewife. She reports problems in recognizing others when meeting them outside the usual context, for example when she meets her parents in the supermarket. HV (male) is 43 years old and teaches writing and coaches in communication training. He experiences severe face recognition problems for as long as he can remember. LW (male) is a 48-year old university professor with longstanding difficulties for example in recognizing colleagues at conferences and students. None of the DPs had a neurological history and their structural MR-scans showed no abnormalities as judged independently by four experienced neurologists. The group of four control subjects was matched with the DP group on age, sex and educational level. All participants gave written informed consent according to the Declaration of Helsinki and the study was approved by the local ethics committee (CMO region Arnhem-Nijmegen, The Netherlands).

### Neuropsychological testing

All participants were presented with an extensive face recognition battery. Visual object recognition and face recognition were assessed with standard clinical tests and additional face and object perception experiments were run in sessions preceding the fMRI measurements. The neuropsychological tests and normative data are described elsewhere [36]. Face matching and face memory were tested with the Benton Face Recognition Test (BFRT) [47] and the Warrington Face Memory Test (WFMT) [48]. We used a computerized version of the latter test to obtain information about speed-accuracy trade-off. Basic visual functions were measured with the Birmingham Object Recognition Battery (BORB) (line length, size, orientation, gap, minimal feature match, foreshortened views and object decision) [49]. To investigate in detail different aspects of face perception, all participants were administered additional face and object perception experiments which have proven useful in previous investigations of face recognition and provided insight in processing strategies in prosopagnosia [4], [17], [36], [43], 50, 51.

Like in our previous studies on prosopagnosia, the behavioral pattern of a normal inversion effects for faces compared to another single object category was measured with the faces and shoes task [17]. Participants were required to select the probe that corresponded with the identity of a simultaneously presented target. The target was always a frontal picture and the two probes underneath consisted of pictures in three quarter profile. Faces and shoes were presented upright and inverted for details, see [17], [50]. Feature-based processing was tested with a part-to-whole matching task which required participants to select the face-part probe (i.e., mouth or eyes) that was the same as that in the simultaneously presented whole face. The same procedure was followed for house-part probes (i.e., door or upper window) that had to be matched to the corresponding part in a whole house stimulus. Faces and houses were presented once upright and once inverted [4], [43]. Participants were instructed to respond as accurately and rapidly as possible. Accuracy and mean response-times were calculated for each test. We compared the accuracy and response times from the upright stimuli with the inverted stimuli in one-tailed paired-sample t-tests. A significantly lower accuracy or longer response time for the inverted stimuli is defined as an inversion effect, whereas a higher accuracy or shorter response time for the inverted stimuli is defined as a paradoxical inversion effect. Data of the control group were normalized and z-scores were obtained for every DP.

### fMRI measurements

#### Stimulus materials

The face and body stimuli were used previously in an fMRI investigation of the neural substrates of processing face and body perception in neurologically intact observers [52]. Pictures of fearful, happy and neutral faces were taken from the Karolinska Directed Emotional Face database [53]. From our own database, pictures of fearful and happy bodily expressions, instrumental (emotionally neutral) bodily expressions (pouring water into a glass) and houses were used. We used houses as stimuli for the control condition, because they constitute a single object category that has been extensively explored in other studies and is known to elicit activation in specific brain areas [24]–[26]. Instrumental body expressions were used because, like emotional expressions, these displays elicit action representation and implicit movement [54], and hence constitute a balanced comparison category for the emotional expressions. All images of faces and bodies were previously validated regarding emotional expression (minimum recognition rate: 75%). For further details concerning the validation procedure, see [52].

A total of 42 images was used, six in every condition (fearful faces, happy faces, neutral faces, fearful bodies, happy bodies, neutral bodies and houses). There was no identity overlap between faces and bodies or between the emotions. Faces were fitted inside a gray oval shape, which masked external aspects of the faces. Body and house stimuli were cut out, removing all background. The faces of the body stimuli were covered with a gray opaque mask. Additionally, one picture of a chair was used as an oddball stimulus. All stimuli were resized to 300 pixels in height and presented on a gray background.

### Procedure

**Figure 1:**
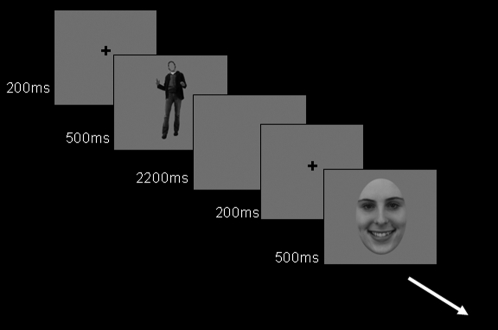
Schematic representation of the experimental design. Participants were instructed to press the response button when a chair was presented.

The design was adapted from our previous study [52]. In order not to exacerbate the face handicap of the DP group, we modified the experimental paradigm from a facial expression categorization task to an oddball detection task thereby also avoiding selective attention to the faces with an emotional expression. Moreover, this procedure excludes that activation profiles are contaminated by motor responses in the conditions of interest while still providing control data on attention to the stimuli. A trial started with the presentation of a fixation cross (200 ms), followed by a stimulus (500 ms) and finally by a gray screen (2200 ms) (see Figure 1). All stimuli were presented six times in random order in an oddball paradigm (participants were instructed to press a response button when a chair was shown). The session consisted of 288 trials (7 conditions×6 identities×6 presentations, plus 36 oddball trials). Additionally, 96 null-events consisting of a gray screen lasting the whole trial length were included to reduce stimulus onset predictability and to establish a baseline [55]. The experiment was preceded by a short practice-session which used a different set of face and body stimuli.

Participants lay supine in the scanner with head movements minimized by an adjustable padded head holder. Stimuli were projected onto a mirror above the participant's head. Responses were recorded via an MR-compatible keypad (MRI Devices, Waukesha, WI), positioned on the right side of the participant's abdomen. A PC running Presentation 9.70 (Neurobehavioral Systems, San Francisco, CA) controlled stimulus presentation and response registration.

### Image Acquisition

Images were acquired using a 1.5 Tesla Sonata scanner (Siemens, Erlangen, Germany). Blood oxygenation level depend (BOLD) sensitive functional images were acquired using a single shot gradient echo-planar imaging (EPI) sequence [TR (repetition time) = 3790 ms, TE (echo time) = 40 ms, 43 transversal slices, ascending acquisition, 2.5 mm slice thickness, with 0.25 mm gap, FP (flip angle) = 90°, FOV (field of view) = 32 cm]. An automatic shimming procedure was performed before each scanning session. A total of 312 functional volumes were collected for each participant. Following the experimental session, structural images were acquired using an MP-RAGE sequence [TR/TE/TI (inversion time) 2250 ms/3.93 ms/850 ms, voxel size 1×1×1 mm].

## Results

### Neuropsychological testing

All DPs scored outside the normal range for the BFRT and/or the WFMT, but none showed an anomalous score on more than one subtest of the BORB suggesting that the visual recognition difficulties of the DPs as measured by these two clinical tests are not due to basic visual perception problems diagnosed in the BORB (see Table 2). AM scored significantly below the mean on the BFRT and WFMT, for both accuracies and response times. HV had a borderline performance on the BFRT and prolonged response times on the WFMT. LW scored within normal range on the BFRT, but on the WFMT both accuracy and response times were anomalous.

**Table 2:** Results from neuropsychological testing.

		Controls	AM	HV	LW
BFRT accuracy (/54)		45.4 (A)	28 (SI)	40 (BL)	44 (A)
WFM accuracy (/50)		44.0	29***	41	34**
WFM RT (ms)		1778	3171***	3853 ***	3171 ***
Faces accuracy (/64)	Upr	63.3	57***	63	64
	Inv	62.0	56**	62	57**
Shoes accuracy (/64)	Upr	62.5	64	64	61
	Inv	62.8	62	64	58**
Faces RT (ms)	Upr	1146	3743 ***	2840 ***	1741**
	Inv	1526	3406 ***	3640 ***	2112
Shoes RT (ms)	Upr	978	2533 ***	1757 ***	1450***
	Inv	1069	2849 ***	1689 ***	1673**
Face-parts accuracy (/64)	Upr	62.7	47***	60*	59**
	Inv	62.0	52***	63	56**
House-parts accuracy (/64)	Upr	62.7	63	62	61
	Inv	63.2	64	64	63
Faceparts RT (ms)	Upr	1562	2099**	4446 ***	3462***
	inv	1755	2224	4130 ***	3229***
Houseparts RT (ms)	Upr	1192	1554*	1703 ***	1917***
	inv	1132	1361	1593 ***	1774***

Response times are shown for correct responses. Comparisons of DP's and matched controls are made by z-scores on the basis of the following control groups:

Control group for the Warrington face memory: N = 25 (18–27 yrs).

Control group for the Faces and Shoes task: N = 11 (18–28 yrs).

Control group for the Face- and Houseparts: N = 21 (18–29 yrs).

Asterisks indicate P-values corresponding to the Z-scores. ^*^ p<.05; ^**^ p<.01; ^***^ p<.001. SI: severe impairment; BL: borderline; A: average.

To measure face and object recognition in a comparable way and assess relative configural processing routines, we compared upright and inverted stimulus matching for each object category [17], [36]. The control group showed an inversion effect for matching faces in both the accuracy (*t*(10) = 1.892, p<.05) and response time (*t*(10) = 3.164, p<.005). The controls showed no inversion effect for matching shoes. For the DPs, the response times were high as previously reported [4], [36]. AM was impaired in matching both upright (Z<−5.75) and inverted (Z<−3.39) faces. Her response times showed a paradoxical inversion effect pattern for matching faces and a normal inversion for matching shoes. HV had accuracies within the normal range, but displayed a normal inversion pattern in the response times for matching faces and a paradoxical inversion effect in the response times for matching shoes. LW showed reduced accuracy for matching inverted faces (Z<−2.82) and inverted shoes (Z<−2.74). His response times for matching upright faces were prolonged (Z>2.39), while the latencies for inverted faces were on average. He displayed the normal inversion pattern for matching faces and shoes in both accuracy and response times.

Feature-based matching was tested with the faces and houses task see [4] for details. The control group showed a normal inversion effect for matching face parts in accuracy (*t*(10) = 1.746, p<.05) and in response time (*t*(10) = 4.754, p<.001). However, they showed a paradoxical inversion effect for matching house-parts in accuracy (*t*(10) = 1.743, p<.05) and response time (*t*(10) = 2.667, p<.01). AM showed lower accuracies for matching both upright (Z = −11.81) and inverted (Z = −5.36) face-parts. Her latencies for matching upright face-parts (Z = 2.51) and house-parts (Z = 2.06) were higher than normal. She displayed a paradoxical inversion effect in the accuracy data for matching face-parts and house parts, and in the response times for matching house-parts. Her response times for matching face-parts showed a normal inversion pattern. HV had a reduced accuracy for matching upright face-parts (Z = −2.00). He also had highly prolonged response times for upright faces (Z = 13.46) and to a lesser extend for inverted faces (Z = 8.27). Latencies for upright houses (Z = 2.28) and inverted houses (Z = 3.31) were also prolonged, but less than for faces. HV showed paradoxical inversion effects in both the accuracy and response times for face-part and house-part matching. LW's accuracy for matching upright (Z = −2.76) and inverted (Z = −3.16) faces was impaired. His responses for matching upright face-parts (Z = 8.87), inverted face-parts (Z = 5.13), upright house-parts (Z = 4.12) and inverted house-parts (Z = 4.61) were prolonged. LW's accuracy data showed a normal inversion pattern for matching face-parts and a paradoxical inversion pattern for matching house parts. He displayed a paradoxical inversion effect in his response times for matching face-parts and house-parts.

### fMRI analysis

All participants performed flawlessly on the oddball detection task.

#### Preprocessing

Imaging data were analyzed using Brainvoyager QX (Brain Innovation, Maastricht, the Netherlands). The first five volumes of each functional run were discarded to allow for T1 equilibration. Pre-processing of the functional data included 3D-motion correction, slice scan time correction, temporal data smoothing (high pass filter 3 cycles in time course) and spatial smoothing with an isotropic 6-mm full-width-half-maximum (FWHM) Gaussian kernel. Images were spatially normalized to Talairach space [56] and resampled to a voxel size of 1×1×1 mm. Statistical analysis was based on the general linear model (GLM), with each condition defined as a separate predictor. Null-events were modeled explicitly.

#### ROI definition

We used a “split-half” method for defining regions of interest (ROI), in order to be sure that the observed effects are not due to a selection bias [57]. The even trials were used to define the ROIs and the odd trials were used for the within ROI analysis. To localize face-sensitive activation in FG, i.e. FFA, we contrasted the even trials of all face conditions (fearful, happy and neutral) with houses (all trials) and identified significant voxels in each subject within a restricted region of the FG (Talairach y-coordinate between −25 and −65). The voxel set comprising this activation determined the ROI, in this case the FFA. The same procedure was followed in a restricted region of the IOG (Talairach y-coordinate <−70). To identify body sensitive areas, we compared the even trials of all bodies (fearful, happy and instrumental) with houses and mapped the selective activation in a restricted region of FG to determine the FBA (Talairach y-coordinate between −25 and −65) and the region around the junction of the middle temporal and middle occipital gyrus to determine the EBA (Talairach x-coordinate between 25 and 60; y-coordinate between −55 and −75; z-coordinate between −15 and 15). We used a liberal threshold (p<.05, uncorrected). Since previous studies reported that cortical face and body selective regions are often weaker or even absent in the left hemisphere [6], [29], we restricted the analysis to the right hemisphere.

**Figure 2:**
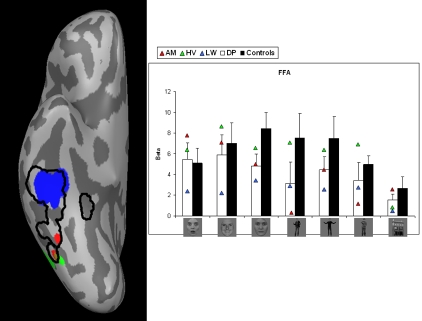
Face-specific activation in right FG when comparing faces (fearful/happy/neutral) with houses. Left: Areas are shown on an inflated right hemisphere. Activation maps of the control subjects are collapsed and displayed by the black contours. Activation of the individual DPs is plotted in color. Right: beta-values by condition, group and DP. Error bars represent one standard error of the mean (SEM). Conditions represent from left to right: fearful faces, happy faces, neutral faces, fearful bodies, happy bodies, neutral bodies and houses. White columns display the average value of the three patients. Black columns show the average value of the controls. Triangles represent the individual values of the DPs.

Smoothed activation maps are projected on the inflated right hemisphere of one subject. For every ROI, the activation maps of the control subjects are collapsed and the result is displayed by the black contours. This procedure allows visualization of the spatial extent of the activation across different subjects. Activation of the individual DPs is plotted in color (see Figures 2 to [Fig pone-0003195-g003] [Fig pone-0003195-g004] 5). The Talairach coordinates of the activation maps are shown in Table 3.

**Figure 3 pone-0003195-g003:**
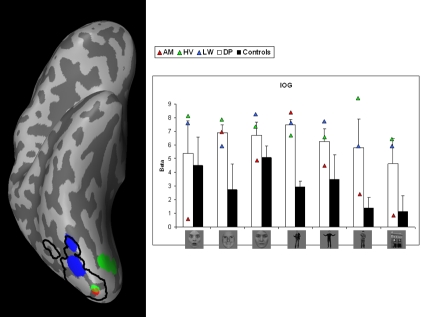
Face-specific activation in right IOG when comparing faces (fearful/happy/neutral) with houses. Left: Areas are shown on an inflated right hemisphere. Activation maps of the control subjects are collapsed and displayed by the black contours. Activation of the individual DPs is plotted in color. Right: beta-values by condition, group and DP. Error bars represent one SEM. Conditions represent from left to right: fearful faces, happy faces, neutral faces, fearful bodies, happy bodies, neutral bodies and houses. White columns display the average value of the three patients. Black columns show the average value of the controls. Triangles represent the individual values of the DPs.

**Figure 4 pone-0003195-g004:**
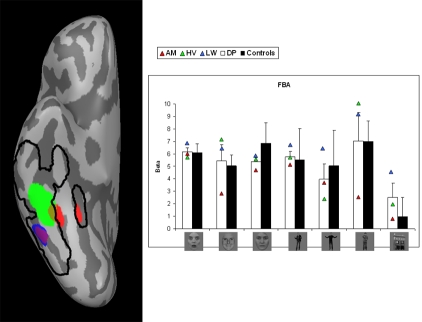
Body-specific activation in right FG when comparing bodies (fearful/happy/instrumental) with houses. Left: Areas are shown on an inflated right hemisphere. Activation maps of the control subjects are collapsed and displayed by the black contours. Activation of the individual DPs is plotted in color. The purple indicates overlap between red (AM) and blue (LW). Right: beta-values by condition, group and DP. Error bars represent one SEM. Conditions represent from left to right: fearful faces, happy faces, neutral faces, fearful bodies, happy bodies, neutral bodies and houses. White columns display the average value of the three patients. Black columns show the average value of the controls. Triangles represent the individual values of the DPs.

**Figure 5:**
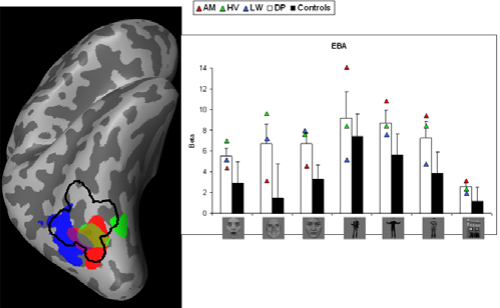
Body-specific activation in right EBA when comparing bodies (fearful/happy/instrumental) with houses. Left: Areas are shown on an inflated right hemisphere. Activation maps of the control subjects are collapsed and displayed by the black contours. Activation of the individual DPs is plotted in color. The purple indicates overlap between red (AM) and blue (LW). Right: beta-values by condition, group and DP. Error bars represent one SEM. Conditions represent from left to right: fearful faces, happy faces, neutral faces, fearful bodies, happy bodies, neutral bodies and houses. White columns display the average value of the three patients. Black columns show the average value of the controls. Triangles represent the individual values of the DPs.

**Table 3:** Number of voxels (N) and Talairach coordinates (range) of ROIs.

Contrast	Mean Controls (range)				AM				HV				LW			
Area	N	x	y	z	N	x	y	z	N	x	y	z	N	x	y	z
(fF+hF+nF)>H																
FFA	427 (9:2208)	40 (31:48)	−42 (−59:−27)	−15 (−33:−5)	24	39	−60	−20	448	36	−64	−9	140	41	−34	−16
IOG	742 (15:2667)	32 (15:55)	−81 (−95:−70)	−7 (−21:9)	9	24	−82	−14	534	25	−74	−13	156	47	−71	−10
(fB+hB+iB)>H																
FBA	1220 (303:2282)	44 (27:58)	−40 (−60:−25)	−15 (−23:−19)	406	34	−49	−9	502	41	−40	−18	34	41	−54	−11
EBA	416 (42:1201)	45 (33:59)	−63 (−74:−52)	4 (−11:15)	737	43	−70	−6	802	36	−66	−10	2404	43	−68	8
fF>nF																
Right AMG	146(146:146)	12(8:16)	−8(−4:−12)	−12(−14:−10)					14	24	0	−8	20	23	0	−7
Left AMG					15	−20	−8	−3	80	−24	1	−5				
hF>nF																
Right AMG	11(0:11)	9(8:9)	−1(−2:0)	−11(−12:−10)									23	23	0	−8
Left AMG	265(0:265)	−13(−18:−11)	−12(−14:−9)	−14(−18:−19)					35	−24	−1	−6				
fB>nB																
Right AMG	258(8:779)	19 (12:26)	−7 (−9:0)	−12 (−17:−7)					142	13	−6	−6				
Left AMG	351 (0:710)	−20 (−28:−11)	−8 (−9:0)	−10 (−19:18)	130	−23	−1	−18	120	−17	−10	−11				
hB>nB																
Right AMG	152 (0:330)	19 (13:25)	−5 (−14:0)	−16 (−21:−7)												
Left AMG	659 (0:819)	−17 (−24:−11)	−4 (−11:0)	−13 (−21:−7)					102	−19	−11	−6				

The coordinate range represents the outer voxels of the collapsed cluster from all controls. Abbreviations: n = neutral; f = fearful; h = happy; i = instrumental; F = face; B = body; H = house; FFA = Fusiform Face Area; IOG = Inferior Occipital Gyrus; FBA = Fusiform Body Area; EBA = Extrastriate Body Area; AMG = Amygdala.

#### Effects of emotional content

The analyses were performed on the beta-values of the odd trials of the conditions. To investigate differences between the DP group and the control group, we used independent samples *t*-tests, corrected for unequal variances (in degrees of freedom).

#### FFA

Figure 2 shows the smoothed face-specific activation (left) and the beta-values of all conditions (right) in FG. The controls show the expected age-dependend higher activation for neutral than for fearful expressions [42]. We calculated the difference between fearful faces and neutral faces and this difference was significantly larger in the control group (*t*(4.946) = −2.583, p<.05). The difference between happy faces and neutral faces was marginally significantly different between groups (*t*(4.906) = −2.051, p<.097). Since previous studies showed a lower activation for faces in DPs compared to controls [11], [15], we used one-tailed post-hoc *t*-tests to compare the activation levels of the three face conditions between both groups. This revealed a marginally significant difference for the neutral faces (*t*(4.980) = 1.929, p<.051).

#### IOG

Figure 3 shows the smoothed face-specific activation (left) in IOG and the beta-values of all conditions (right). A *t*-test on the difference between fearful faces and neutral faces showed no significant difference between both groups (*t*(4.510) = .0233, p<.826). The difference between happy faces and neutral faces was also not significantly different between the DPs and controls (*t*(4.989) = −1.235, p<.272).

#### FBA

Figure 4 shows the smoothed body-specific activation (left) and the beta-values of all conditions (right) in FBA.The difference between either fearful bodies (*t*(4.475) = −.088, p<.934) or happy bodies (*t*(4.567) = .321, p<.762) and instrumental bodies was not significantly different between both groups.

#### EBA

Figure 5 shows the smoothed body-specific activation (left) and the beta-values of all conditions (right) in EBA.The difference between fearful bodies and instrumental bodies was not different between groups (*t*(3.786) = 1.153, p<.317). A *t*- test on the difference between happy and instrumental bodies revealed no significant between-group difference (*t*(3.722) = .339, p<.573).

#### Effects of categorical selectivity

To investigate the selectivity of processing faces and bodies in the brain, we calculated the difference between the mean of the three face conditions and the mean of the three body conditions in FFA and IOG. A comparison using *t*-tests showed that this difference was smaller in the control group in IOG, but it did not reach statistical significance (*t*(3.961) = 2.122, p<.102). We also calculated the difference between the mean of all body conditions and the mean of all face conditions in FBA and EBA. Independent sample *t*-tests showed no significant between-group differences.

#### Processing of neutral faces

Since the main body of research on DP concerns neutral faces, we compared the activation level of neutral faces between both groups in all four ROIs, using *t*-tests. In addition to the above mentioned difference in FFA, this revealed a marginally significantly higher activation for neutral faces in EBA in the DP group (*t*(4.955) = 2.044, p<.097).

#### Effects of emotion in amygdala

Finally, we performed a post-hoc analysis, in which we defined the amygdala in each subject, based on the individual anatomy. This ROI consisted in each hemisphere of a cube of 13×13×13 voxels around the center of the amygdala and we performed a second GLM in this area. The results are shown in Table 3. Contrasting fearful faces with neutral faces revealed significant activation in all three patients (left amygdala in AM; bilateral amygdala in HV and right amygdala in LW). Comparing happy with neutral faces showed activation in two patients (left amygdala in HV and right amygdala in LW). Fearful compared with neutral bodies differentially activated the amygdala in two patients (left amygdala in AM and bilateral amygdala HV). Happy bodies triggered significantly more amygdala activity in one DP (left amygdala in HV) compared to neutral bodies.

## Discussion

The first major finding is that compared to the control group, the DP group displays a similar activation level for the emotional faces, but a lower activation in FFA for neutral faces. A lower activation level in DP for neutral face perception in FG is consistent with earlier reports [11], [15]. The present results are compatible with the theoretical perspective on face recognition difficulties argued for previously [18], [21] suggesting a higher threshold for neutral face recognition performance in prosopagnosics. This relative difficulty with neutral faces is based on the notion that faces are more difficult stimuli than many other categories they are routinely compared with.

Emotional stimuli trigger a higher level of arousal e.g. [58], [59] and emotion in a face constitutes an additional feature that carries important communicative information and is therefore more salient. This saliency hypothesis is supported by a number of behavioral studies, with different visual tasks, that have demonstrated that adding emotional information to a face results in a greater tendency to capture attention [60]–[63]. Note though that the emotion effects we observe are not specific for emotions with a negative valence since we obtain similar effects for both fearful and happy (although less pronounced) expressions.

However, normal FFA activation for facial expressions in the presence of lower than normal activation for neutral faces suggests that the activation boost is triggered more in he emotion processig than in the impaired face processing system in ventro-temporal cortex. Studies on perception of emotional faces in normals have hypothesized the existence of a feedback mechanism between FG and amygdala [38], [39], [64]–[66]. The possibility that such feedback connections from the amygdala may be active in prosopagnosia and boost face processing was already suggested in an earlier study of emotional faces in prosopagnosia [43]. Two acquired prosopagnosics were presented with both a neutral and emotional part-to-whole face matching task. The patients had lesions in FG and/or IOG, but the results showed normal activation in other face-sensitive area's (amygdala, superior temporal sulcus), for the contrast between emotional and neutral faces. The patients were also more accurate and faster when they performed the task with emotional faces compared to neutral ones. Moreover, the patients showed a normal inversion effect for matching emotional but not for neutral faces.

Lower neural activity in the DPs for neutral faces, but not for emotional faces is compatible with a dual route model of face perception as argued first in de Gelder and Rouw [4] and adapted in de Gelder et al. [43], involving subcortical structures along a pathway that is able to proces facial expressions (the pulvinar-superior colliculus-amygdala route) [67] which in turn may boost face representations in the cortical route in temporal cortex even when face representations in temporal cortex are weak as shown by the lower activation for neutral faces in the DP group [43]. The pattern observed here is in line with this and may also explain why emotional content facilitates the cortical processing of faces in prosopagnosia. Consistent with this, we observed a higher activity level of the amygdala for emotional faces compared to neutral ones. A related and more extreme phenomenon is observed in hemianopic patients, who are unable to consciously report the presentation of a face in the blind visual field and do not show FG activation when presented facial expressions in the blind field but who perform well above chance in tasks where they have to guess the facial expression [68].

Our second main finding concerns the categorical specificity of face vs. body representation in DPs. We compared the activation of body conditions in the face selective regions and of the face conditions in the body selective regions between both groups. On the one hand, our findings indicate that perceiving neutral faces results in a higher activation of EBA in the DP group, compared to the control group. Combined with the lower activation for neutral faces in FFA, this increased activation in EBA might indicate an anomalous cerebral processing route in DP. It may be the case that (neutral) faces are processed in the areas more dominantly dedicated to body perception. On the other hand, we find a higher activation for perceiving bodies in IOG. These combined findings indicate that the neural correlates of perceiving faces and bodies, as manifested in IOG and EBA show a lower degree of specificity in DP.

For body triggered activity we find no difference in neutral vs. emotional expressions between both groups, either in FBA or EBA. This indicates that the anomalous neuro-functional substrate in our DP group for neutral faces does not extent to the processing of bodies and bodily expressions. This is in line with recent behavioral data showing no impairment in recognizing neutral body postures in one DP patient [35]. One of the DPs (HV) in the present study participated in a previous ERP study on perception of neutral faces and neutral bodies [36] and the results of both studies are partly converging. Righart & de Gelder [36] measured the electrical brain correlates of the inversion effect as an index of configural processes (the ability to perceive stimuli as one configuration as opposed to an assemblage of features [69]). HV differed significantly from the control group in face processing on two accounts. He displayed a paradoxical ERP inversion effect (the reverse pattern from the controls) around 100 ms after stimulus presentation (P1 amplitude) and no inversion effect around 170 ms after stimulus presentation (N170 latency). But his results for bodies did not differ from the controls.

An important and relevant difference between face and body perception concerns the coding of identity. A face contains all necessary information about the identity of a person and we are used and trained to recognize identity by the face. A person can be readily identified on the basis of his face, but identification based on the body alone is far less evident. The different pattern in FG for faces and bodies may therefore reflect the possibility that FG is more involved in processing person identity [7] which is typically more based on the face than on the body.

Notwithstanding the well documented involvement of FG in face perception, its precise role of FG in prosopagnosia is still a matter of debate. We do not clearly understand at present how factors like maturation of different cortical areas, like the FG, are important for normal face recognition. Reduced volume of the right temporal lobe has previously been reported in a DP patient [70]. A structural imaging study in six DP subjects investigated volumetric and morphometric properties in occipito-temporal cortex and showed a decreased volume of the FG that correlated with face recognition deficits [71]. At the neuro-functional level, recent data collected from normals show a correlation between the volumetric size of the right FFA and recognition memory for neutral faces [72]. This study also investigated the development of category specific brain areas and the results suggest that the relative size of the FFA increases during development. Moreover, the development of the FFA takes longer compared to that of object selective areas (lateral occipital complex) or face sensitive areas in the superior temporal sulcus see [73] for review and discussion. These findings support the notion that DP may be associated with abnormal development of FG which may be either a consequence or a cause of anomalous face skills. Lesions in acquired prosopagnosia (AP) patients often include the FG e.g. [43], [74], although other cases have also been reported with lesions more posterior than the face sensitive part of the FG e.g. [75], [76]. Besides the heterogeneity across lesion localization in AP, considerable heterogeneity consists in behavioral symptoms in DP [77]. Since successful face-processing is likely to involve a variety of hierarchical and parallel processes, impairments in different processes will result in different types of behavioral and neuro-anatomical correlates. The results from the present study clearly demonstrate the importance of emotional information in face processing and urge (future imaging) studies to take the modulatory effect of emotion into account, in order to further untangle the complex nature of DP.
